# Comment on: The Canadian International Medical Graduate Bottleneck: A New Problem for New Doctors

**Published:** 2012-03-31

**Authors:** Robert Obara

**Affiliations:** Trinity College, University of Dublin, Ireland

Re: *The Canadian International Medical Graduate Bottleneck: A New Problem for New Doctors*.^1^ As a Canadian studying medicine abroad (CSA), I see first-hand the ever-growing number of students like myself facing an uphill battle to return home. There were an estimated 650 CSAs in Ireland in 2010,^2^ and since then we have seen class sizes (and number of CSAs) grow further.

The process of returning to Canada to complete our residency training varies by province, and there exists an irony in the process that is no more evident than in British Columbia (BC). Only 24 of 279 residency positions in BC were dedicated to international medical graduates (IMGs) in the 2012 match,^3^ but virtually no BC residents in their final year of medical school abroad were able to apply for these first iteration positions. This is due to a restrictive BC assessment program for IMGs, that is simply timed too late in the year, such that most CSAs are ineligible to apply to the match in their final year of medical school; the current IMG system is better suited for landed immigrants who hold foreign medical degrees.

The Dean of Medicine at the University of British Columbia (UBC), Dr. Gavin Stuart, stated in an interview, “clinical skills are probably one of the big areas of difference of Canadian graduates from those that are graduating from Ireland or Australia… those individuals may have comparable knowledge base, [but] have not had the clinical exposure.”^4^ Meanwhile, BC is actively recruiting physicians from the very countries where the CSAs are studying.^5^ Health Match BC has been making recurrent trips to Ireland and the UK, to recruit only foreign doctors but not Canadians finishing their medical degree abroad.

Unless there are significant changes to better allow CSAs to return home upon graduation, more and more students will find themselves unable to serve Canada.
